# Lifestyle intervention in BRCA1/2 mutation carriers: study protocol for a prospective, randomized, controlled clinical feasibility trial (LIBRE-1 study)

**DOI:** 10.1186/s40814-016-0114-7

**Published:** 2016-12-19

**Authors:** Marion Kiechle, Christoph Engel, Anika Berling, Katrin Hebestreit, Stephan Bischoff, Ricarda Dukatz, Wolf-Dieter Gerber, Michael Siniatchkin, Katharina Pfeifer, Sabine Grill, Maryam Yahiaoui-Doktor, Ellen Kirsch, Uwe Niederberger, Nicole Marter, Ute Enders, Markus Löffler, Alfons Meindl, Kerstin Rhiem, Rita Schmutzler, Nicole Erickson, Martin Halle

**Affiliations:** 1 Department of Gynecology and Center for Hereditary Breast and Ovarian Cancer, Klinikum rechts der Isar, Technical University Munich (TUM), Ismaninger Str. 22, 81675 Munich, Germany; 2 Institute for Medical Informatics, Statistics and Epidemiology, University of Leipzig, Haertelstrasse 16-18, 04107 Leipzig, Germany; 3 Department of Prevention and Sports Medicine, Klinikum rechts der Isar, Technical University Munich (TUM), Ismaninger Str. 22, 81675 Munich, Germany; 4 Institute for Nutritional Medicine, University Hohenheim, Fruwirthstr. 12, 70593 Stuttgart, Germany; 5 Institute for Medical Psychology and Sociology, University Hospital Schleswig-Holstein, Campus Kiel, Preusserstr. 1-9, 24105 Kiel, Germany; 6 Center for Hereditary Breast and Ovarian Cancer, University Hospital Cologne, Kerpener Str. 34, 50931 Cologne, Germany; 7 Else Kroener-Fresenius Prevention Center, Klinikum rechts der Isar, Technical University Munich (TUM), Ismaninger Str. 22, 81675 Munich, Germany

**Keywords:** BRCA1, BRCA2, Hereditary breast cancer, Hereditary ovarian cancer, Lifestyle intervention

## Abstract

**Background:**

Women with highly penetrant BRCA mutations have a 55–60% lifetime risk for breast cancer and a 16–59% lifetime risk for ovarian cancer. However, penetrance differs interindividually, indicating that environmental and behavioral factors may modify this risk. These include lifestyle factors such as physical activity status, dietary habits, and body weight. The modification of penetrance by changing lifestyle factors has not thus far been investigated in a randomized trial in BRCA mutation carriers.

**Methods:**

Therefore, we intend to enroll 60 BRCA1/2 mutation carriers in a pilot feasibility study (Lifestyle Intervention Study in Women with Hereditary Breast and Ovarian Cancer (LIBRE) pilot). This multi-center, prospective, controlled trial aims to randomize (1:1) participants into a (1) multi-factorial lifestyle intervention group (IG) versus (2) the control group with usual care (CG). The primary endpoint is feasibility and acceptance of a structured interdisciplinary lifestyle intervention program over 12 months (at least 70% of the patients to complete the 1-year intervention). Furthermore, the effects on physical fitness, BMI, quality of life, and stress coping capacity will be investigated.

During the first 3 months, women in the IG will receive structured, individualized and mainly supervised endurance training of ≥18 MET*h/week (MET = metabolic equivalent task) and personal nutritional counseling based on the Mediterranean diet. During the subsequent 9 months, the IG will receive monthly group training sessions and regular telephone contacts for motivation, whereas the CG will only receive usual care (one general counseling on healthy nutrition and benefits of regular physical activity on health status). At randomization and subsequent time points (3, 6, 12 months), cardiopulmonary fitness will be assessed by spiroergometry and nutritional and psychological status by validated questionnaires.

**Discussion:**

This pilot study will investigate the optimal strategy to improve physical fitness, nutritional habits, and psychological factors in women at high risk for developing breast or ovarian cancer. The results of this pilot feasibility study will be the basis for a larger prospective randomized trial including clinical events (LIBRE).

**Trial registration:**

ClinicalTrials.gov, NCT02087592

## Background

Tumor suppressor genes, such as breast cancer (BRCA)1 and 2, are important regulators for suppressing tumor development. Women with highly penetrant BRCA mutations have a lifetime risk of 55–60% for breast cancer and 16–59% for ovarian cancer [1, 2]. Penetrance rates vary because of endogenous factors, such as gene polymorphisms, as well as exogenous factors, such as the number of pregnancies, year of birth, and physical activity during youth [3, 4]. The risk for breast cancer is lower if genotype carriers were born before 1940, gave birth, or were physically active during their youth [5, 6].

Prospective studies have revealed that regular physical activity can significantly reduce breast cancer incidence in post- and premenopausal women, the risk being reduced on average by 25% [7]. Furthermore, the risks of recurrence and mortality in women with breast cancer are reduced by 50% if they engage in regular physical activity [8]. Further advantages of physical activity include a gain in quality of life, increased fitness, and improved tolerance of chemotherapy [9].

Nutrition also influences the risk of breast cancer. Obesity and weight gain increase the risk of breast cancer in both pre- and postmenopausal subjects [10, 11]. A weight gain of more than 20 kg after the age of 18 doubles the risk of breast cancer. Furthermore, women with a body mass index (BMI) of >30 kg/m^2^ have a greater risk of developing distant metastases and early mortality [12]. In a prospective study with sporadic breast cancer on patients under adjuvant standard therapy, a calorie and fat-reduced nutrition program led to a significant reduction in recurrence rates [13].

Further risk factors for breast cancer include depression, a pessimistic outlook on life, and problems in coping with stress [14, 15]. Numerous studies have overwhelmingly documented the substantial significance of an optimistic life perspective for different psychological and somatic disorders [16]. A positive association was shown between an optimistic outlook on life and psychological well-being, health, stress reduction, and mortality, as well as improved recovery rates [17–19].

So far, no studies have been performed prospectively assessing lifestyle intervention in women with hereditary breast cancer or women with a deleterious BRCA mutation. Even retrospective data analysis is rare. There is currently only one publication on this subject by Manders et al. in 2011 [20], which reports on an association between increased body weight and an increased risk for breast cancer in BRCA1/2 mutation carriers.

Therefore, we aim to perform a large randomized intervention trial assessing whether a long-term multifactorial lifestyle intervention program, including (1) a structured physical endurance training, (2) nutritional counseling stressing the Mediterranean dietary pattern, and (3) stress coping strategies, will lead to a reduction in breast cancer incidence and mortality in BRCA1 and 2 mutation carriers (Lifestyle Intervention Study in Women with Hereditary Breast and Ovarian Cancer (LIBRE)). Before this large trial can be started, we will execute a feasibility study (LIBRE-pilot), which is outlined here. This smaller trial with 60 patients will evaluate whether women will be compliant and will sufficiently adhere to a demanding intervention program. Based on the results of this first pilot trial, we aim to set up a subsequent larger trial to investigate the efficacy of the intervention on clinical endpoints. The present paper describes the objectives and design of the feasibility trial, hereafter shortly referred to as “LIBRE-1” (Lifestyle Intervention Study in Women with Hereditary Breast and Ovarian Cancer, 1 = pilot).

## Methods

### Trial design

**Fig. 1:**
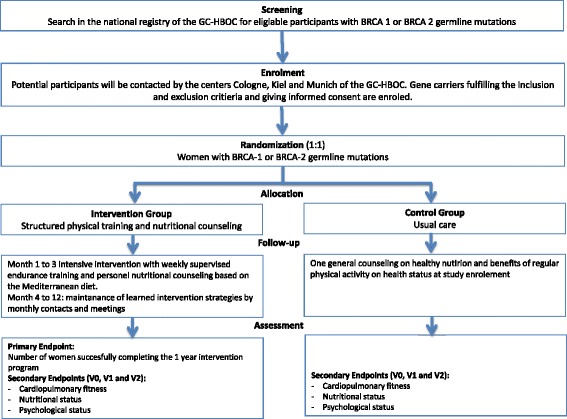
Design of the trial

LIBRE-1 will be a multicenter, prospective, two-armed randomized (1:1) controlled clinical trial including *n* = 60 BRCA1 or 2 carriers. The aim of the study is to evaluate adherence to and acceptance of a structured, 1-year exercise program combined with a Mediterranean dietary pattern in BRCA mutation carriers. Additionally, physical fitness, body weight, quality of life, and stress coping capacity will be assessed. The study design is outlined in Fig. 1 and includes four visits (study entry (SE), start of intervention (V0), 3 months after start of intervention (V1), and 12 months after start of intervention (V2)). The schedule of enrollment, interventions, and assessments is shown in Table 1.

**Table 1:** Schedule of enrolment, interventions, and assessments

	Enrolment	Start	3 months	6 months	9 months	12 months	24 months	36 months	+12 months
Visit	SE	V0	V1	V1–6	V1–9	V2	V3	V4	Follow-up
Enrolment									
Eligibility screen	X								
Informed consent	X								
Randomisation	X								
Interventions									
Intervention group (I)	X	X	X	X	X	X	X	X	X
Control group (C)	X	X				X	X	X	X
Assessments									
Clinical baseline	C + I								
Clinical follow up						C + I	C + I	C + I	C + I
Spiroergometry	C + I		C + I			C + I			
Accelerometry	C + I		C + I	C + I	C + I	C + I			
Questionnaire “training” (IPAQ-L)		C + I	C + I	C + I	C + I	C + I	C + I	C + I	
Questionnaire “nutrition” (MEDAS, EPIC-FFQ)	C + I		C + I	I	I	C + I	C + I	C + I	
Lab	C + I		C + I			C + I	C + I	C + I	
Clinical examination	C + I		C + I			C + I	C + I	C + I	
Anthropometry	C + I		C + I			C + I	C + I	C + I	
Psychological questionnaires	C + I		C + I			C + I	C + I	C + I	

### Participants

The goal is to recruit a minimum of 60 patients for the feasibility study. Recruitment and study conduct take place at three centers in three different regions in Germany (Munich, Kiel, and Cologne). Patients will be recruited from the German Consortium Hereditary Breast and Ovarian Cancer (GC-HBOC), a national registry for BRCA1 and 2 carriers. BRCA1- or 2-positive participants have been included in this surveillance program before and are contacted on a regular basis regarding clinical events. They have previously received genetic analysis mainly because of an amplified family history for breast cancer. They have received additional counseling before and are aware of their cancer lifetime risks. Table 2 details the study’s inclusion and exclusion criteria. Contacting the carriers for potential inclusion in the LIBRE-1 study will be done by the local study centers which are familiar with the patient history. Patients will either be contacted by telephone or during their regular visits to the hospital. Information regarding the LIBRE-1 trial will be given in person by physicians involved in the GC-HBOC program and previously instructed regarding the LIBRE-1 study. If patients agree to participate in the trial, they will have to sign consent forms and, if eligible, will be enrolled into the trial.

**Table 2:** Study population: inclusion and exclusion criteria

Inclusion criteria (all criteria must apply): • Women with a pathogenic BRCA1 or BRCA2 germline mutation • Age between 18 and 69 years • Written informed consent	
Exclusion criteria: • Metastatic tumor disease • Expectancy of life <3 years • Limited cardiovascular and lung diseases (instable CHC, heart failure stage IV, COPD GOLD IV, maximal blood pressure at rest >160/100 mmHg) • Significant orthopedic problems, not allowing exercise • Serious diseases, not allowing a participation in group interventions (e.g., psychiatric or internal ailment) • Karnofsky status scale <60% • Women with VO_2_ max > 150% of general population • Women with an exercise capacity <50 W • Food allergies not allowing consumption of a Mediterranean dietary pattern • Vegans • BMI < 15 kg/m^2^ • Pregnancy • Insufficient knowledge of the German language • Current participation in other lifestyle intervention trials	

### Intervention

#### Intervention group

The intervention program group is designed to last 12 months. The first 3 months includes intensive (regular) contact with physical activity and nutritional education sessions. The learned strategies will then be maintained and monitored in the following 9 months through monthly contacts and meetings. The lifestyle intervention program comprises the following measures:

1. *Physical activity*: The LIBRE training program is primarily an endurance-oriented training, which will be completed in the course of 1 year. After a mandatory introductory lecture on the theory behind the intervention training, the goal is to increase physical activity to ≥18 MET*h/week (MET = metabolic equivalent task). One MET is equivalent to resting energy expenditure, so multiple METs express exercise intensity that is performed over a certain time, e.g., hours per week. 18 MET*h/week is approximately equivalent to brisk walking (4.5 MET) for 4 h per week. This activity level has been correlated consistently with a reduction of morbidity and mortality in breast cancer patients [8, 21]. This goal should be achieved within the first 12 weeks and maintained throughout the whole study period. Each subject will receive an individual training plan, which will continuously be adapted according to her fitness status. The training plan is divided into two phases: the intensity of the initial phase, weeks 1–6, should be at least 50–60% of the peak oxygen consumption (VO_2_ peak), and the optimization phase, weeks 7–12, 60–75% of the VO_2_ peak. In the first 12 weeks of the intervention program, training will take place twice a week as supervised training and once a week as home-based training (HBT). Afterwards, supervised training units will only be carried out monthly, so that training in months 4–12 will be mainly HBT. Training will continue to take place with an intensity of at least 60–75% of the VO_2_ peak in accordance with the individual training plan.
  A record of compliance with the training intervention program as well as the achieved MET*h/week will be kept in the training diaries (questionnaire on physical activity, V0–V2). Participants should record their daily activities as well as intentional physical activity including intensity and duration of training. The training intensity levels will then be assessed by spiroergometry and outlined in the diary, which facilitates training control. Monthly supervised training units (as of V1) offer the opportunity to realign training intensity and discuss any problems with adherence to training. In addition, physical activity is to be recorded using questionnaires (International Physical Activity Questionnaire (IPAQ) [22, 23] from V0 to V2), motion sensors (accelerometer from SE to V2), and a physical fitness assessment by cardiopulmonary exercise testing by spiroergometry (VO_2_ peak) [24] at time points SE, V1, and V2.
2. *Nutrition*: Within the framework of the LIBRE-1 study, the nutrition intervention is based on the principles of a Mediterranean dietary pattern (MD). Furthermore, obese patients (BMI ≥ 35 kg/m^2^) will be instructed to limit their energy (kcal) intake. Nutrition intervention in the IG consists of an intensive phase of 3 months in line with the intensive physical exercise program. During this phase, bi-weekly nutrition education group courses will be held by dietitians. They include a cooking class and guided tour of a supermarket. Afterwards, nutrition courses will be reduced to monthly intervals. The main objective of the nutrition intervention is to provide practical nutritional training, which should enable the subjects to achieve a long-term change in their eating habits, replacing it with the MD. Eating habits will be recorded using validated questionnaires (EPIC-Food Frequency Questionnaire (FFQ) and Mediterranean Diet Adherence Screener (MEDAS)) at the time points SE, V1, and V2. The questionnaires are described below in the “Measurements” section.
3. *Psychological support*: Explicit psychological support is not planned. Psychological support of the IG will comprise solely of an explanation of the psychological data survey questionnaires given to the study participants. The participants will be informed that the objective of the lifestyle study is, among others, that the lifestyle change including regular physical activity and healthy eating should lead to an improvement in general life quality, stress reduction, and a more optimistic outlook on life. In order to verify this, the participants will receive questionnaires (Screening Scale for Chronic Stress (SSCS), Life of Orientation Test-Revised (LOT-R), European Organisation for Research and Treatment of Cancer (EORTC) Quality of Life Questionnaire Core 30 and Breast Cancer Module 23 (QLQ-C30/-BR23), and Assessment of physical activity and nutrition (BKAE)) at several time points (SE, V0, V1, V2). Advice on the psycho-oncological aspects of the LIBRE study will be given during the introduction lecture at the beginning of the study. The psychological advice given to the study participants will serve to inform the subjects of the significance and improvement of psycho-social lifestyles for the prevention of breast and ovarian cancer, as well as to promote compliance and recognize possible psychological impediments for participation in the study.

#### Control group

The CG will receive a mandatory introduction lecture on the positive effects of physical activity on the incidence and prognosis of breast cancer. Afterwards, all participants will be given a brochure providing the most important facts on this topic. In contrary to the IG, no training and no physical activity diaries will be provided. Changes in physical activity behavior are however to be measured identically to the interventional group by questionnaires (IPAQ from V0 to V2), motion sensors (accelerometer SE–V2), and examinations of physical exercise capacity (VO_2_ peak) at the time points SE, V1, and V2. Additionally, a dietitian-led group lesson on healthy eating will be held for the CG/UC. During this lesson, the subjects will receive general information based on the recommendations of the German Society of Nutrition, which is referred to as “usual care in Germany” in this study. Eating habits will be recorded identically to the intervention group via validated questionnaires (EPIC-FFQ and MEDAS questionnaire) at the defined time points SE, V1, and V2.

Finally, the control group will receive an explanation of the psychological data entry forms, given to the study participants. An explicit psycho-oncological intervention strategy is also not intended in this group. The participants will be informed that changes in daily routine concerning physical activity and dietary habits among other things should result in the improvement of quality of life, a reduction of stress, and a more optimistic approach to future life. In order to verify this, the participants will receive questionnaires (SSCS, LOT-R, EORTC QLQ-C30/-BR23, LIBA, and BKAE) at SE, V0, and V2. The psychological information of the CG/UC corresponds to those of the IG.

### Measurements

In order to achieve the study goals and to answer the research questions, structured questionnaires and interview sheets, diaries, clinical and instrument-based examinations, and blood and stool tests will be used.

#### Medical history

Data will be gathered by means of an interview at SE. In addition to a basic clinical assessment, pre-existing internal and gynecological illnesses and general risk and protective factors such as body weight, alcohol consumption, nutritional behavior, level of activity, and number of pregnancies will be assessed.

#### Sociodemographic

Data will be collected at SE. The psychosocial and socioeconomic risk and protective factors such as living conditions, job situation, and income level will be assessed.

#### Medical radiation exposure

Data will be gathered by means of an interview at SE. The previous diagnostic and therapeutic X-ray exposure will be documented.

#### Study satisfaction questionnaire

This questionnaire will be handed out at V1 and V2. Questions are intended to capture the subject’s acceptance of the study, their satisfaction with the supervision, an evaluation of the physical activity and nutrition program, and their assessment of everyday practicality of the intervention program, where relevant.

#### Cardiovascular assessment

Within this examination at time points SE, V1, and V2, the patient’s medical history will be taken and a clinical examination with focus on the cardiovascular system will be performed. A resting electrocardiogram (ECG), pulmonary function testing, and blood pressure measurements are obligatory. Resting heart rate and blood pressure are to be recorded as measuring parameters.

#### Exercise testing/spiroergometry

Spiroergometry is an objective and well-known examination assessing cardiopulmonary exercise capacity. As the maximal oxygen uptake (VO_2_ max) and the aerobic and anaerobic respiratory capacity will be measured, the results enable a precise statement about the maximum cardiopulmonary capacity as well as aerobic and anaerobic capacity. The following target parameters (VO_2_ peak and O_2_ at ventilator anaerobic threshold (VAT)) [24] provide information about the fitness level at visits SE, V1, and V2.

#### Accelerometer

The AiperMotion 440/500 is a recognized physical activity meter, which objectively measures the scope of activity, as well as the steps and speed of movement [25–27]. The device is light (72 g) and has a three-dimensional digital acceleration sensor, which with an accuracy of 95%, classifies the level of movement into the categories “active,” “slow walk,” “fast walk,” and “jog.” The average level of activity is recorded as a physical activity level (PAL) value. The movement sensors will be distributed at five different time points in the first study year (SE–V2).

#### International Physical Activity Questionnaire

The IPAQ comprises five areas of activity, which are examined independently of each other [28]. The questionnaire is a simple instrument, which can be used to collect internationally comparable data for health-promoting physical activity over the last 7 days. The IPAQ is to be assessed at all study time points (SE–V2).

#### Examination of body composition

The examination of physical constitution comprises the documentation of anthropometric parameters such as height; body weight; waist, hip, and upper arm measurements; skin fold tests; and the bioelectric impedance analysis (BIA measurement) at the visits SE, V1, and V2, respectively. These measurements serve to record the percentage of body fat and the BMI and to determine level of lean body mass.

#### Blood tests

Within the context of the study, various blood values will be regularly determined (Table 3) in both the study arms at the time points (SE, V1, V2).

**Table 3:** Blood measurements

Routine blood parameters measured in recruiting centers: • GPT (ALT) • Total cholesterol • HDL-cholesterol • LDL-cholesterol • Triglycerides • Glucose • Blood cell count • HbA1c	
Blood parameters measured in central or special laboratories: • Insulin • 25-OH-vitamin D • Selen • ß-carotine • Proencephalin A 119 D159 (Sphingotec GmbH) • Proneurotensin 1–117 (Sphingotec GmbH) • hsCRP (high sensitivity CRP) • Omega-3, 6, and 9 fatty acid content in the membrane of erythrocytes	

#### Stool samples

All subjects will provide stool samples (SE, V1, V2). A collective microbiome and metabolome analysis will be carried out at a later date.

#### EPIC-FFQ [29–31]

The questionnaire refers to food consumption during the last year and covers 148 food items. For each item, questions are asked concerning the average quantity consumed (pre-defined servings) and the frequency of consumption (1–6 times per day, week, month, or year). Color photos simplify the definition of serving sizes for food items, which are not consumed in normal household quantities. Furthermore, respondents are specifically asked about their use of cooking fats/oils, the frequency of consumption of sauces with meat and fish, the fat content of the milk they consume, the use of sugar and milk in coffee and tea, and the seasonal consumption of fresh fruit and vegetables. The data input and evaluation of the questionnaires is to be carried out via the study management system (SMS) for health research, which has been developed and is supervised by the Department of Epidemiology of the German Institute of Human Nutrition Potsdam-Rehbruecke (DIfE). The EPIC-FFQ will be conducted at SE, V1, and V2 in both study arms.

#### Mediterranean Diet Adherence Screener [32]

The MEDAS questionnaire was developed within the scope of the PREDIMED study. The evaluated English version was translated for the LIBRE-1 study and complemented by pictures of serving sizes (cf. CRF MED). The German version of the MEDAS will be validated within the scope of the pilot study. The MEDAS questionnaire is to be conducted at SE, V1, and V2 in both study arms.

#### Measurement of quality of life (EORTC QLQ-C30/-BR23) [33, 34]

In order to acquire health-related quality of life (HRQOL), a composed questionnaire, a 30-item core questionnaire (QLQ-C30), and a 23-item breast cancer module (QLQ-BR23) will be used before and after the intervention period [35]. The advantage of the QLQ-C30/-BR23 includes the present-day relevance and the previous validation for breast cancer (QLQ-BR23). Validation studies in European countries and the United States have been conducted in cross-cultural context [36, 37].

#### Measurement of optimism (life orientation test, ten items, LOT-R by Glaesmer et al. [38])

The LOT-R was developed to collect individual differences of generalized optimism versus pessimism in the form of a personality variable. It has been applied in various intervention studies, which, among other things, look at the consequences of dispositional optimism on behavior, emotion, and physical health. We hypothesize that regular physical activity and the implementation of healthy nutrition (IG) can lead to a more optimistic attitude to life.

#### Measurement of chronic stress [39]

The SSCS by trier inventory on chronic stress is a 12-item (short version) questionnaire, which measures five different aspects of chronic stress: lack of social recognition, pressure of business, social stress, chronic anxiety, and overstrain [39].

#### Measurement of attitudes and views on physical exercise and healthy eating (BKAE, 18 items)

One of the main intentions of the present LIBRE study is to investigate the practicability of the intervention program in the sense of good compliance of mutation carriers towards an exercise program and Mediterranean diet. In order to understand how different factors may influence compliance, measurements of subjective parameters are needed (such as attitudes towards a behavior, subjective norms, and perceived behavior control), which represent a necessary link between objective factors (age and other demographics, stage of disease, comorbid conditions, treatments, stress pressure, daily hassles, etc.) and intentions related to behavior as well behavior itself (doing sports and keeping a specific diet). According to the theory of planned behavior (see Fishbein and Ajzen [40]), the intentions related to behavior result from an interplay between attitudes towards a behavior, subjective norms, and perceived behavior control. The theory of Fishbein and Ajzen was applied in the last years in many studies which dealt with health psychology (e.g., surgery intentions, food adherence, and physical activity behavior among others) and represents one of the well-proven ways to investigate subjective mechanisms of compliance [41–44]. In the context of the Fishbein and Ajzen [40] model, the study may answer the following questions: (1) Which are the predictors of regular participation in the preventive measures (exercise, nutrition)? (2) To which degree are attitudes, subjective norms, or perceived behavior control towards a behavior relevant for the intention of behavior (i.e., to train regularly and to eat healthy) and for the de facto behavior shown?

### Study endpoints

The primary endpoint of the study is the number of randomized women who successfully complete the first 3 months of the intervention program and the monitoring/maintenance phase after 1 year. The study is considered feasible if at least 70% of the patients fulfill the criteria (see Table 4 for details).

**Table 4:** Criteria for successful completion

Attendance of 70% of the planned lifestyle intervention events (training sessions and nutrition courses) within the first 3 months • Conduct of study visit after 1 year • No withdrawal of informed consent within 1 year • No new occurrence of any of the exclusion criteria during the first 3 months	

Secondary endpoints of the study include the measurements of quality of life (EORTC QLQ-C30-/BR23), stress coping (SSCS), optimism grade (LOT-R), body mass index (BMI), eating habits, nutrient and fat calorie intake (EPIC-FFQ), adherence to a Mediterranean diet (MEDAS), maximal oxygen intake (VO_2_ max), and physical activity (IPAQ). These measurements will be performed after 3, 6, and 12 months of intervention and compared to the baseline measurements at SE.

### Statistical analysis and randomization

The sample size for the feasibility study is to be at least 60 women. For this study, the sample size was not determined based on statistical assumptions and tests, but on practical grounds that each center should recruit 20 participants. Primary and secondary endpoints will be analyzed descriptively. The primary endpoint will be determined according to the criteria shown in Table 4. Secondary endpoints will be described using absolute or relative frequencies, means with standard deviations, or medians with inter-quartile ranges as appropriate, for each of the study arms at the different time points. Measurements at months 3, 6, and 12 will be compared and tested against the baseline values using either parametric or non-parametric tests as appropriate. Randomization is to take place using envelopes, which will be deposited at the study center before the study begins. The group allocation (randomization) will be done centrally, using randomly permuted blocks of length 2–6. The randomization ratio is to be 1:1. We will use participating center and previous breast cancer as stratification factors. Each center will receive consecutively numbered, opaque envelopes, unique to them, split into two sets (one for previously diseased and one for healthy participants), containing the results. For each new participant, the principal investigator in the center will choose and open the first envelope from the diseased or healthy batch accordingly. As the study is not blinded, the allocation result will be concealed neither from participants nor from the principle investigators.

## Discussion

To the best of our knowledge, the LIBRE-1 study is the first prospective randomized lifestyle intervention trial in BRCA mutation carriers worldwide. Since the patients in the intervention arm will be given a strict and intensely structured intervention program, the purpose first is to assess the feasibility of the study design in a multicenter setting including 60 female mutation carriers. We are aware that the study protocol is time-consuming and demanding for the patients including regular exercise and changes in nutritional habits but believe it necessary to cover/investigate all relevant factors. Especially, during the first study visit, the questionnaires amount to a large contribution for the participants. However, the questionnaires can be answered during the waiting time between the examinations, e.g., spiroergometry, ECG, and body composition, lasting approximately half a day. In order to meet a high compliance rate, all participants will be informed beforehand about the time necessary for study examinations. For the time spent for examinations, patients will also receive a small financial reimbursement.

The Mediterranean diet though is widely accepted as a healthy diet in Germany. In addition, randomized controlled intervention studies have revealed that it is effective in the primary prevention of cardiovascular diseases [45], in improving diabetes [46] and cognitive function [47], and recently also in the primary prevention of invasive breast cancer [48].

Once compliance and acceptance of the study protocol have been proven (LIBRE-1), we will proceed with a larger cohort of mutation carriers with the intention to investigate whether the improvement of physical fitness, body weight, quality of life, and stress coping capacity leads to a reduction of breast cancer risk, progression of disease, and even mortality. On the basis of the LIBRE-1 results, it will be possible to outline a lifestyle program with high adherence even over 12 months of intervention (LIBRE trial). The current feasibility study will also reveal barriers to and constraints of the intervention, which will then have to be modified for the larger clinical trial.

One of the foreseen limitations of the trial will be selection bias for those patients being interested in lifestyle intervention and for those who will be randomized to usual care, especially for highly motivated participants. The latter patients may be disappointed on being assigned to the control arm and might either withdraw from the study or follow exercise instructions from the IG. These biases cannot be excluded in any lifestyle intervention trial. However, physical activity will be assessed using questionnaires and an accelerometer in both arms, and any cross-over of patients can be monitored. In addition, physical fitness will be objectively assessed by spiroergometry in both arms, which will also yield information on the long-term involvement of physical activity.

On the other hand, restraints for less motivated patients, e.g., due to professional obligations or as cares for children or other family members, may have an impact on adherence to supervised exercise sessions and everyday physical activities. In addition, a cross-over from CG to IG group for the Mediterranean dietary pattern cannot be excluded as previously outlined for physical activity. However, nutritional habits will also be assessed by questionnaire and BMI and body composition measured in both groups. Because of these problems in lifestyle intervention trials, it is a clear aim to avoid cross-over or bias of investigations by assuring that patients be treated identically regardless of the group assignment.

This LIBRE initiative (pilot and clinical study) will have the potential to reveal whether a structured lifestyle intervention program may prevent tumor incidence in BRCA mutation carriers. This will have to be compared with current risk-reducing strategies for women at risk, including prophylactic mastectomy, and could then be added to early detection programs. Furthermore, by following a structured exercise program and dietary changes, mutation carriers will attain an independent and self-governed role in cancer prevention and successfully reduce their stress levels [39]. By improving and optimizing the latter, we expect a preventive health effect not only on a somatic but also on the mental status of these patients.

## Abbreviations

BIA: Bioelectric impedance analysis
BMI: Body mass index
BRCA: Breast cancer gene
CHC: Coronary heart condition
COPD: Chronic obstructive pulmonary disease
ECG: Electrocardiogram
GC-HBOC: German Consortium Hereditary Breast Ovarian Cancer
HBT: Home-based training
IPAQ: International Physical Activity Questionnaire
LIBRE: Lifestyle Intervention Study in Women with Hereditary Breast and Ovarian Cancer
LOT-R: Life of Orientation Test-Revised
MD: Mediterranean dietary pattern
MET: Metabolic equivalent task
QLQ-C30/-BR23: Quality of Life Questionnaire Core 30 and Breast Cancer Module 23
SE: Study entry
SSCS: Screening Scale for Chronic Stress
V: Visit
